# Optimizing Outcomes in Gastrointestinal Stromal Tumors: A Surgeon’s Perspective

**DOI:** 10.7759/cureus.71771

**Published:** 2024-10-18

**Authors:** Mena Louis, Jerrell Fang, Brian Gibson

**Affiliations:** 1 General Surgery, Northeast Georgia Medical Center Gainesville, Gainesville, USA; 2 Trauma and Acute Care Surgery, Northeast Georgia Medical Center Gainesville, Gainesville, USA

**Keywords:** gastrointestinal stromal tumors, gist management, surgical resection, tumor surveillance, tyrosine kinase inhibitors

## Abstract

Gastrointestinal stromal tumors (GISTs) typically originate in the stomach (60%-70%), followed by the small intestine (20%-30%), with less frequent occurrences in the colon, rectum, and esophagus. The location of the tumor significantly affects both its clinical presentation and treatment approach. Gastric GISTs generally have a better prognosis, while tumors in the small intestine or rectum are associated with a higher likelihood of aggressive growth and recurrence.

Surgical resection is the cornerstone of treatment for localized GISTs, with the aim of achieving a complete (R0) resection with negative margins. Preserving tumor integrity during surgery is critical, as rupture could lead to peritoneal spread and worsen outcomes. Minimally invasive surgery may be an option for smaller tumors in favorable locations, while larger or more complex cases may require open surgery.

In addition to surgery, tyrosine kinase inhibitors are integral to the treatment of GISTs, especially in cases where the tumor is unresectable, metastatic, or at a high risk of recurrence. Agents such as imatinib have revolutionized GIST treatment, offering neoadjuvant therapy to shrink tumors prior to surgery and adjuvant therapy to reduce recurrence risk after surgery. Long-term monitoring with regular imaging is essential, particularly in high-risk patients, due to the potential for late recurrences. Familiarity with these management strategies is vital for optimizing patient outcomes in GIST care.

## Introduction

Gastrointestinal stromal tumors (GISTs) are the most common mesenchymal tumors of the gastrointestinal tract, representing a distinct entity within the spectrum of soft tissue sarcomas [1]. Arising from the interstitial cells of Cajal, or their precursors, GISTs can occur anywhere along the gastrointestinal tract, with a predilection for the stomach and small intestine [2]. These tumors are characterized by their unique molecular and genetic profiles, particularly mutations in the *KIT* gene or platelet-derived growth factor receptor alpha (*PDGFRA*) gene, which drives their pathogenesis [3]. The identification of these mutations has revolutionized the diagnosis and treatment of GISTs, distinguishing them from other mesenchymal tumors and providing a targeted therapeutic approach [4].

The clinical presentation of GISTs can vary widely, depending on the size and location of the tumor [5]. Patients may present with nonspecific symptoms such as abdominal pain, gastrointestinal bleeding, or an abdominal mass, while others may remain asymptomatic until the tumor is discovered incidentally in imaging studies [6]. The diagnosis of GIST is typically confirmed through a combination of imaging modalities, endoscopic evaluation, and histopathological analysis, with immunohistochemistry playing a crucial role in distinguishing GISTs from other tumors [7]. CD117 (*KIT*) positivity is a hallmark of GISTs, with DOG1 also serving as a sensitive marker [8,9]. Additionally, risk stratification based on tumor size, location, and the mitotic rate is essential for guiding management and predicting prognosis [10].

The management of GISTs has evolved significantly over the past two decades, largely due to the advent of tyrosine kinase inhibitors (TKIs) such as imatinib [11]. Surgery remains the cornerstone of treatment for localized disease, with the goal of achieving complete resection with negative margins [12]. However, the introduction of TKIs has transformed the approach to both resectable and unresectable GISTs, offering neoadjuvant and adjuvant options that have improved survival outcomes [13]. Despite the progress made, high-risk and advanced GISTs remain difficult to manage, making ongoing research and a multidisciplinary approach essential for improving patient outcomes [14].

## Case presentation

**Figure 1:**
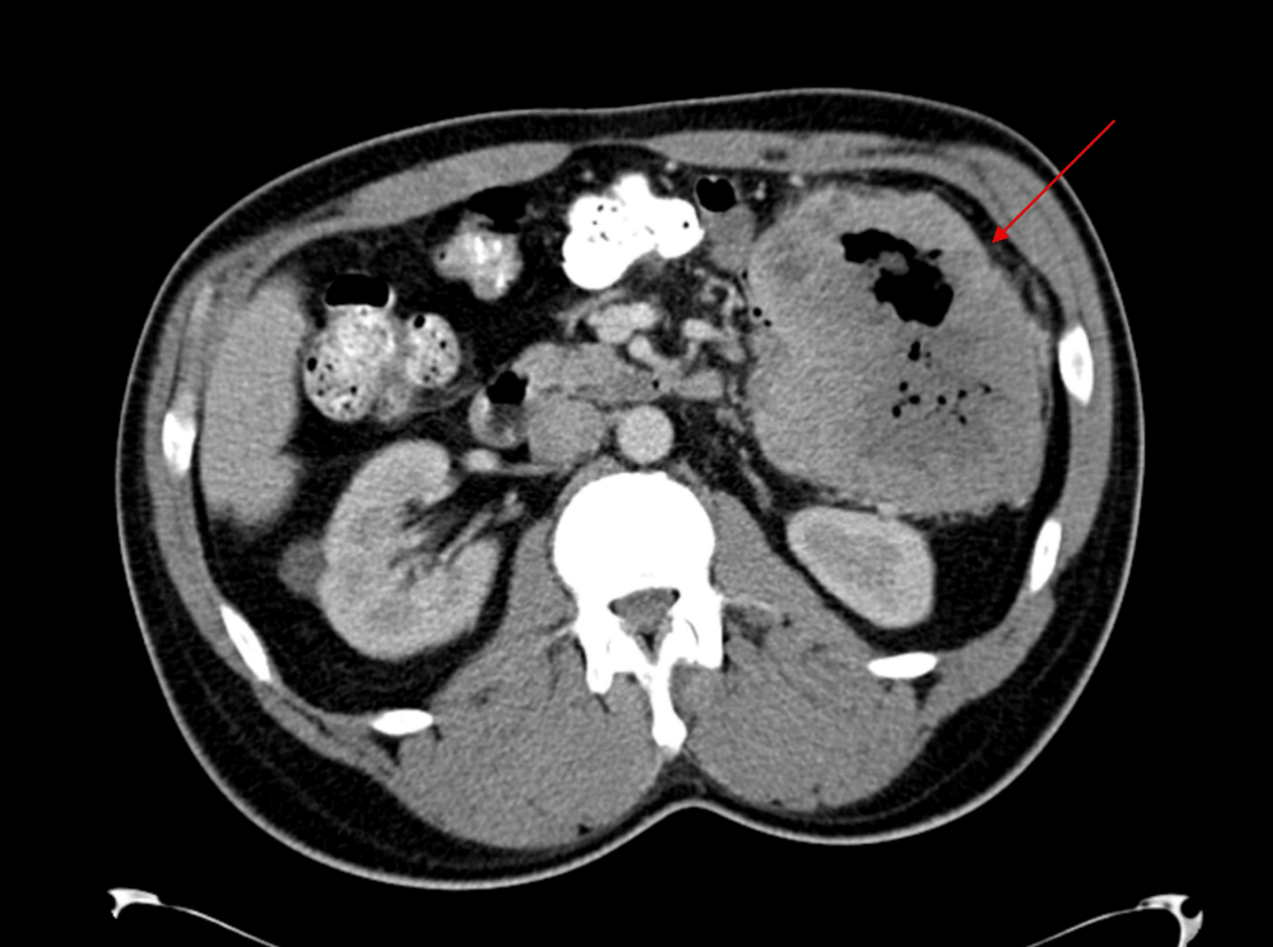
Axial view of contrast-enhanced CT of the abdomen and pelvis showing a large left-sided abdominal mass measuring 11.7 x 11.7 x 10.3 cm (red arrow), closely associated with the small bowel and descending colon.

**Figure 2:**
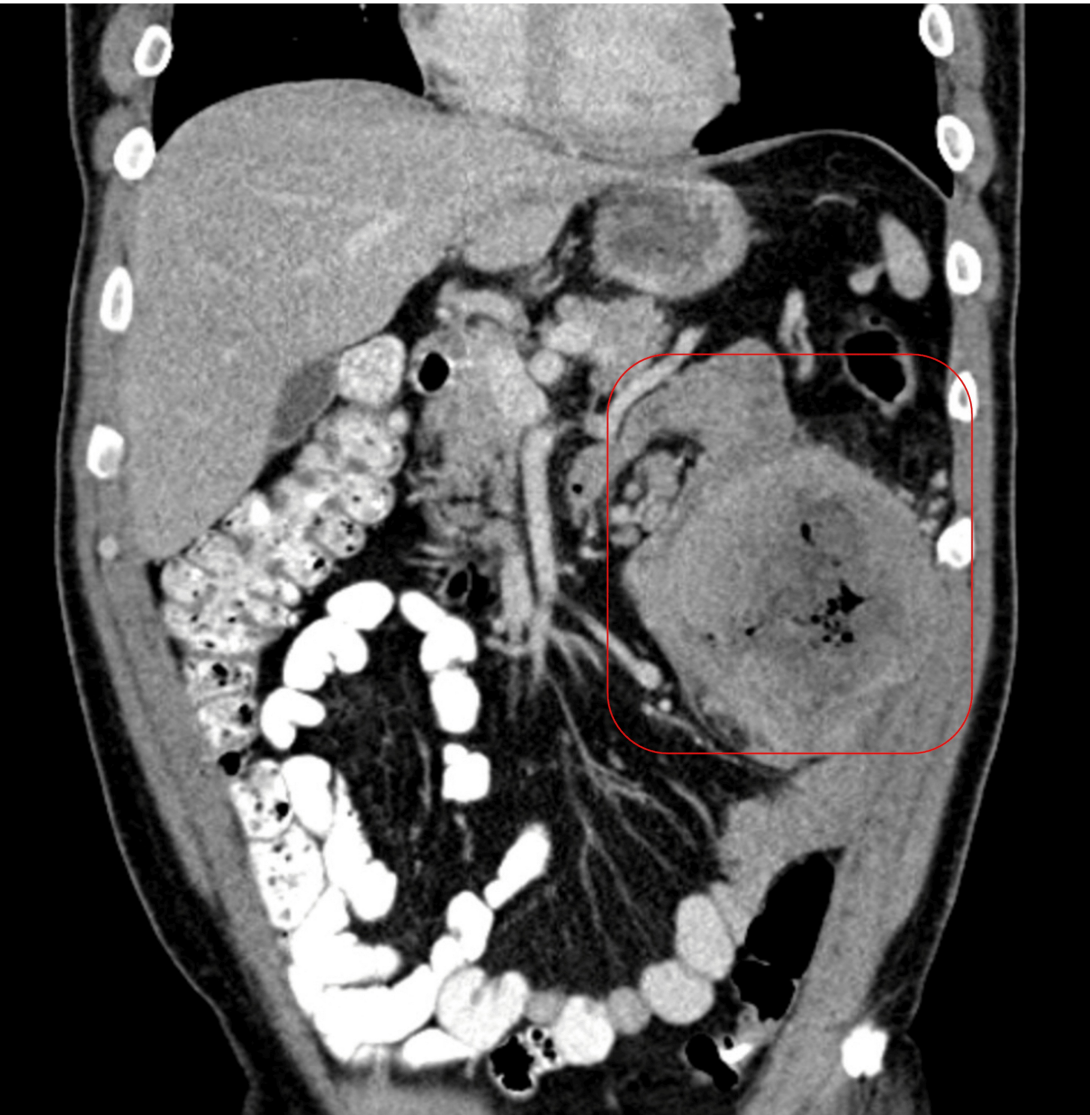
Coronal view of contrast-enhanced CT of the abdomen and pelvis showing a left-sided abdominal mass measuring 11.7 x 11.7 x 10.3 cm (red square).

A 54-year-old male with a history of atrial fibrillation and obstructive sleep apnea, managed with continuous positive airway pressure, and deep vein thrombosis in his right lower extremity presented with progressive left upper quadrant abdominal pain, accompanied by unintentional weight loss of approximately 85 pounds over several months. The pain was sharp, aching, and rated 9 out of 10 in severity, worsening after meals. Despite using pain medication as needed, the pain persisted, prompting further evaluation. A CT scan of the abdomen and pelvis revealed a large left-sided abdominal mass measuring 11.7 x 11.7 x 10.3 cm, closely associated with the small bowel and descending colon, with central low attenuation and air pockets suggestive of malignancy or abscess (Figures 1, 2). Mild left-sided hydronephrosis was noted, likely due to compression of the left ureter by the mass. Further investigation confirmed a diagnosis of GIST through histopathological analysis of a biopsy, revealing significant nuclear atypia, extensive necrosis (95%), and a high mitotic rate of 24 mitoses per 5 mm², classifying the tumor as high-grade.

Due to the tumor's size, location, and symptomatic nature, the patient was started on targeted therapy. The targeted therapy in this case was imatinib, a TKI, commonly used in the treatment of GISTs, particularly those with mutations in the receptor tyrosine kinase (*KIT*) or *PDGFRA* genes.

**Figure 3:**
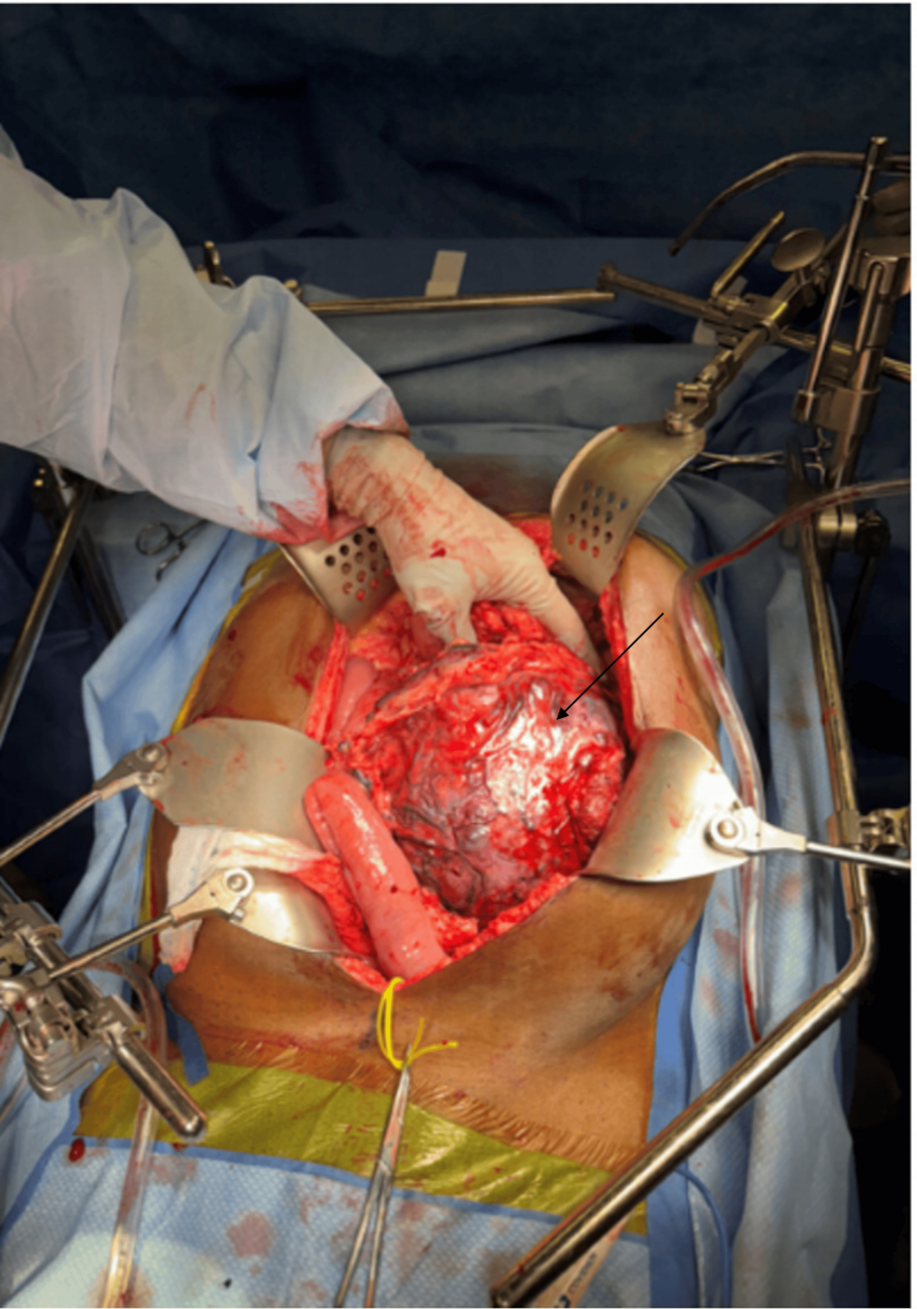
Intraoperative image showing a large intra-abdominal mass (black arrow) invading multiple loops of the small bowel, the left colon, and the abdominal wall.

Despite this, he continued to experience severe abdominal pain, necessitating surgical intervention. An exploratory laparotomy was performed, resulting in en bloc resection of the retroperitoneal mass, including segments of the small bowel, descending colon, and part of the left abdominal wall (Figure 3). The pathology report following resection confirmed the presence of a 20-cm high-grade GIST with significant necrosis and a close margin of 0.2 cm from the nearest peritoneal margin, though the bowel mucosal margins were negative for neoplasia. Postoperatively, the patient's recovery was uneventful, and he was discharged with plans for ongoing follow-up and management.

Ongoing follow-up and management for this patient typically involve regular imaging (CT or MRI) to monitor for recurrence, especially within the first five years post-surgery, when the risk is highest. Adjuvant therapy with imatinib, a TKI, is also recommended, especially for patients with high-risk GISTs. Standard practice involves administering imatinib for at least three years postoperatively to reduce the risk of recurrence. In some cases, where the risk of recurrence is particularly high, therapy may be extended beyond three years. Follow-up also includes regular monitoring of liver function and other parameters to manage potential side effects of the therapy.

## Discussion

GISTs are the most common mesenchymal tumors of the gastrointestinal tract, with a distinctive molecular pathogenesis, primarily driven by mutations in the *KIT* or *PDGFRA* genes [3]. These mutations lead to the constitutive activation of tyrosine kinase receptors, driving the growth and survival of GIST cells [3]. Although rare, GISTs account for approximately 1%-2% of all gastrointestinal malignancies, and their management requires a thorough understanding of their biology, diagnosis, and treatment strategies [15].

GISTs are predominantly characterized by mutations in the *KIT* gene (75%-80% of cases) or the *PDGFRA* gene (5%-10% of cases) [3]. These mutations lead to uncontrolled cell proliferation and tumor growth, making these pathways critical targets for therapy [13]. Diagnosis of GIST is confirmed through imaging and histopathological examination, and CD117 (*KIT*) positivity is the hallmark of GISTs, with DOG1 serving as an additional sensitive marker [16,17]. The differential diagnosis should consider other spindle cell tumors, and molecular testing for *KIT* and *PDGFRA* mutations is essential for both diagnosis and therapeutic planning [18].

Risk stratification in GIST is based on tumor size, mitotic rate, and anatomical location [10]. The National Institutes of Health (NIH) consensus criteria and the Armed Forces Institute of Pathology (AFIP) criteria are commonly used to predict the risk of recurrence [19]. Tumors larger than 5 cm with a high mitotic rate (>5 mitoses per 50 high-power fields) are considered high risk, warranting aggressive treatment and close follow-up. Tumor location also plays a role, with gastric GISTs generally having a better prognosis compared to those located in the small intestine or rectum [7].

Surgery remains the cornerstone of treatment for localized GISTs [4]. The primary goal is to complete resection with negative margins (R0 resection) [12]. Minimally invasive techniques, such as laparoscopic resection, are increasingly used for smaller tumors, particularly in the stomach, but open surgery may be necessary for larger or more complex tumors [20]. Achieving clear margins is crucial, as incomplete resection is associated with a high risk of local recurrence [21]. Lymphadenectomy is generally not required, as lymph node metastasis is rare in GIST [22].

Imatinib, a TKI, is the first-line treatment for GISTs with *KIT* or *PDGFRA* mutations [23]. Neoadjuvant therapy with imatinib is often used in cases where the tumor is large or involves critical structures, allowing for tumor downsizing and more effective surgical resection [24]. Adjuvant imatinib is recommended for patients with high-risk GISTs, typically administered for three years post-surgery [25]. The duration of therapy may be extended in cases with particularly high recurrence risk [25]. For patients with imatinib-resistant GISTs, alternative TKIs such as sunitinib and regorafenib are available [26].

Metastatic GISTs are primarily managed with systemic therapy, as surgical options are limited. Imatinib remains the first-line treatment, with dose escalation considered in cases of disease progression [24]. Sunitinib and regorafenib are used as second- and third-line treatments, respectively [27]. Recent advances have introduced newer TKIs, such as avapritinib and ripretinib, for patients with specific mutations or resistance to earlier lines of therapy [28]. For select patients with limited metastatic disease, surgical resection of metastases may be considered, particularly if the primary tumor is well-controlled [29].

Patients with resected GIST require long-term follow-up due to the risk of late recurrence [6]. Surveillance typically involves periodic imaging, with the frequency and duration tailored to the initial risk of recurrence [30]. High-risk patients may require more frequent follow-up, especially during the first five years post-resection, when the risk of recurrence is highest [21].

Ongoing research into the molecular mechanisms underlying GISTs has led to the development of novel therapeutic agents and strategies [14]. Immunotherapy, targeting specific molecular alterations, and combination therapies are areas of active investigation [31]. Additionally, understanding the resistance mechanisms to current TKIs may provide insights into overcoming therapeutic challenges in GIST management [3].

## Conclusions

The management of GISTs requires a comprehensive approach that integrates precise surgical techniques with targeted therapies. Achieving complete resection with negative margins remains the cornerstone of treatment for localized GISTs, while the use of TKIs has revolutionized the management of unresectable or high-risk tumors. Given the potential for late recurrence, long-term surveillance is essential. Surgeons must be well-versed in the complexities of GIST to optimize patient outcomes, ensuring that each case is managed according to the latest evidence-based practices.
